# Hospitals and the Doctrine of "Cy-Pres"

**Published:** 1910-04-30

**Authors:** 


### Institutional Law Points.

### HOSPITALS AND THE DOCTRINE OF "CY-PRES."

It is sometimes said that when the lawyers are in merry mood they drink the health of the man who makes his own will; yet the home-made will is by no means the only kind of testamentary document which is a source of emolument to the legal profession. It constantly happens that a testator who is charitably disposed has his will drawn in the most careful manner, yet when the time comes to put it into operation it is found impossible to carry out his intentions. The difficulty arises in many cases from the reluctance of those who are responsible for the administration of charitable bequests to interfere in any way with the designs of the testator. If the governors of a hospital hear that Mr. A. B. is going to make a will which is to benefit their institution, they do not presume to interfere. They do not care to look a gift horse in the mouth. The lawyer may do his part well enough, but it is not always considered necessary to have regard to the views of those who are in the habit of administering hospital charities. In the result we have disputes between trustees as to how the money shall be applied, appeals to the Charity Commissioners, applications to the Court, application of the doctrine of *cy-près*, and last, but not least, costs of all parties out of the estate.

### The Weir Hospital Case.

The Weir Hospital case, which was decided lately by Mr. Justice Eve, affords an interesting example of the difficulties which may arise even in connection with a scheme which is apparently well thought out and considered.

The material facts may be briefly stated. Mr. Benjamin Weir, who died in 1902, left over £100,000 upon trust for "a dispensary, cottage hospital, convalescent home, or other medical charity," to be called the Weir Hospital, for the benefit of the inhabitants of Streatham and neighbourhood. He left his freehold residence known as the "Hawthorns," Balham, to be used as a hospital, and directed that the moneys should be expended in maintaining this place as a hospital. He also left another house—No. 12 Devonshire Road—to be used in conjunction with the "Hawthorns." The annual income came to about £3,000 a year. The trustees, upon consideration, came to the conclusion that it was impracticable to carry out the trust in its entirety in that the site provided was inadequate for the establishment of a hospital with so large an endowment. They decided, however, to comply with the testator's request to establish a medical charity at the "Hawthorns" by turning it into a nursing home. They also decided to turn No. 12 Devonshire Road into a dispensary. As the income of the endowment was more than sufficient for these purposes they decided to devote not more than £50,000 to enlarging and supporting the Bolingbroke Hospital, which should in future be called the "Weir and Bolingbroke Hospital," arrangements being made so that the people of Streatham should have a voice in the management of the hospital. After a local inquiry had been held, the Charity Commissioners approved a scheme giving effect to the above proposals. Certain of the trustees, however, appealed to the Court from the decision of the Commissioners.

### What is the Doctrine of "Cy-Pres."

It was alleged that the scheme really involved a breach of trust, and could not be sanctioned even in accordance with the doctrine of *cy-près*. It may be mentioned that that doctrine, stated in two words, means that a charitable gift is not to be allowed to fail merely because there may be some uncertainty as to the particular institution which the testator intends to benefit. Mr. Justice Eve held that the nurses' home established by the scheme was, in his opinion, a medical charity within the meaning of the testator's will, and that the appropriation of the "Hawthorns" to that institution did not involve any breach of trust. He held also that the fact that there is surplus revenue raises a case for the application of the *cy-près* principle. It was also contended that to devote part of the revenue to an outside hospital was to fail to give effect to the testator's intention to benefit Streatham; but the learned judge thought that this argument failed to give effect to the fact that the testator intended to benefit "Streatham and neighbourhood." In the result, therefore, he upheld the view of the Charity Commissioners, and allowed the scheme to go through.

### How it Affects Hospitals.

This case is an example of the way in which a gift for the purposes of a hospital charity may prove difficult to apply in practice. From the abundance of his generosity the testator provided more than enough to turn his old home into a hospital and supply it with the necessary funds, and the problem then arose as to how the surplus should be applied. As it seems to us, the scheme adopted by the majority of the trustees, which has now been sanctioned by the Court, is in every respect desirable. This is not the first time that the curious doctrine of *cy-près* has been applied for the benefit of hospitals. It proceeds from notions adopted from the Roman and civil law, which are very favourable to charities, that legacies given to public uses not ascertained shall be applied to some proper object. In a case heard in 1778 a testator gave his residue as follows: One-half to the Foundling Hospital, and the other half to the Lying-in Hospital; and if there should be more than one of the latter, then to such of them as his executor should appoint. He died without having appointed an executor. Lord Thurlow upheld the gift of the second half, and directed an inquiry as to which of the lying-in hospitals it should be paid.

